# Forensic investigations of disasters: Past achievements and new directions

**DOI:** 10.4102/jamba.v15i1.1490

**Published:** 2023-09-15

**Authors:** Irasema Alcántara-Ayala, Ian Burton, Allan Lavell, Anthony Oliver-Smith, Alonso Brenes, Thea Dickinson

**Affiliations:** 1 Institute of Geography, National Autonomous University of Mexico (UNAM), Mexico City, Mexico; 2 Faculty of Geography and Planning, School of the Environment, University of Toronto, Toronto, Canada; 3 Latin American Faculty of Social Sciences, San José, Costa Rica; 4 Department of Anthropology, University of Florida, Gainesville, Florida, United States; 5 Dickinson and Associates, Toronto, Canada

**Keywords:** root causes, risk drivers, forensic investigations of disasters, FORIN, social construction of risk, disaster risk creation and construction, transformational change, existential threats, new world order

## Abstract

In the 2020s, understanding disaster risk requires a strong and clear recognition of values and goals that influence the use of political and economic power and social authority to guide growth and development. This configuration of values, goals, power and authority may also lead to concrete drivers of risk at any one time. Building on previous disaster risk frameworks and experiences from practice, since 2010, the ‘Forensic Investigations of Disasters (FORIN)’ approach has been developed to support transdisciplinary research on the transformational pathways societies may follow to recognise and address root causes and drivers of disaster risk. This article explores and assesses the achievements and failures of the FORIN approach. It also focuses on shedding light upon key requirements for new approaches and understandings of disaster risk research. The new requirements stem not only from the uncompleted ambitions of FORIN and the forensic approach but also from dramatic and ongoing transformational changes characterised by climate change, the coronavirus disease 2019 (COVID-19) pandemic and the threat of global international confrontation, among other potential crises, both those that can be identified and those not yet identified or unknown.

**Contribution:**

Disasters associated with extreme natural events cannot be treated in isolation. A comprehensive “all risks” or “all disasters” approach is essential for a global transformation, which could lead to a better world order. To achieve this, an Intergovernmental Panel for Disaster Risk is suggested to assess risk science periodically and work towards sustainability, human rights, and accountability, within a development and human security frame and on a systemic basis and integrated perspective.

## Introduction

### Seeking to understand and manage risk

The dominant paradigm for some time has been that disaster risk can be described as a function of the hazard events themselves, the level of exposure to the events and the vulnerability of the people and property exposed. This has been summarised as:


Risk=ƩHazard×Exposure×Vulnerability or simply R=H×E×V. [Eqn 1]

In recent years, and especially now in the second decade of the 21st century, all three of the elements in the equation are changing rapidly, many in increasingly uncertain ways. One reason for this is climate change, which is increasing the magnitude and frequency of hydro-meteorological hazard events. Under the previous paradigm, although major floods, tropical cyclones, droughts and other atmosphere-related extreme events were understood to be large if not entirely ‘natural’, today the hazard component is increasingly interpreted as a social construction related to the degrading relations between society and the natural environment (Lavell 1996). In Latin America since the 1990s, such hazards have been referred to as socio-natural (Fernández 1996; Lavell 1996). Now climate change and associated increased variability have consolidated the idea of the socio-natural and social construction of hazards. Thus, hazards themselves are now being substantially changed for the worse by anthropogenic climate change and increasing complexity and concatenation (Cardona et al. 2012; Lavell et al., 2012).

Turning to exposure, one notes the continued growth of exposure of people and their assets and the built environment despite greater knowledge of where the hazard events will occur. Scientific knowledge has expanded, and societies as a whole have become wealthier, and this has often led to a greater sense of confidence or over-confidence about the human capacity to control nature. It is clear, however, that the coronavirus disease 2019 (COVID-19) pandemic and climate change have recently revealed how obsolescent this view is.

A third component is vulnerability. Growth in inequality affects vulnerability in both more and less developed societies. The irony is that while the overall inequality of wealth between nations has decreased, the inequalities within nations have increased. Within countries, the gap between more and less vulnerable people is growing, and the more vulnerable or poorer people are more likely to live in or be relocated to hazardous areas (Lavell & Maskrey 2014).

**FIGURE 1:**
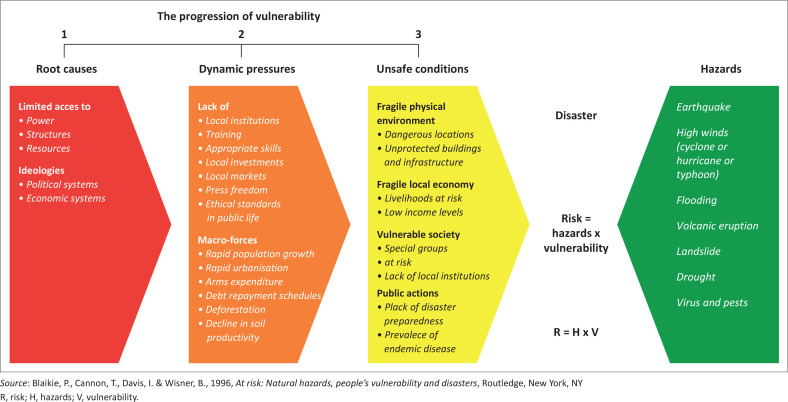
Pressure and release model: the progression of vulnerability.

The project known as FORIN (Forensic Investigations of Disasters) has sought since 2010 to identify root causes of disaster risk and thus disaster, in the interest of clarifying those social structures and forces and the related institutional and social actors that fuel and invigorate the drivers of vulnerability and exposure which, when interacting with hazard, produce a disaster (Oliver-Smith et al. 2016). FORIN, was formulated as a distinct approach (Burton 2010; Integrated Research on Disaster Risk 2011), which also integrates the strengths of earlier disaster risk frameworks, particularly the pressure and release (PAR) model (Blaikie et al. 1996; Wisner et al. 2004) (Figure 1) and builds on lessons learned in practice to support research on the transformational pathways that societies must follow to recognise and address root causes and drivers of disaster risk.

**FIGURE 2:**
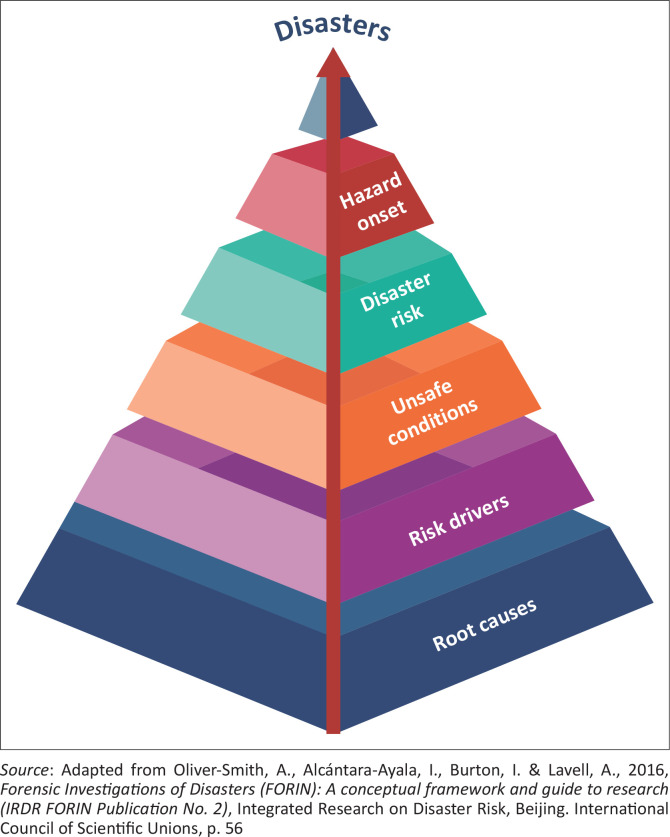
From the social construction of risk to the social production of disaster.

‘Forensic Investigations of Disasters (FORIN): a conceptual framework and guide to research’, was published also in Beijing by the IRDR-IPO. Later a Spanish translation was published (Investigación Forense de Desastres [FORIN]: un marco conceptual y guía para la investigación) by the National Autonomous University of Mexico, and recently a version was published in Chinese (FORIN – 灾害风险取证研究) (Oliver Smith et al. 2016). FORIN highlighted a fundamental problem hindering disaster risk management, namely the absence of recognition of social, economic, political and cultural underlying causes. The processes of risk construction were seen to comprise a more profound and complex chain of interactions and interdependencies among root causes, risk drivers, unsafe conditions, vulnerability, exposure and hazard occurrence (Figure 2).

Unsafe conditions (exposure to hazards) are shaped through a series of disaster risk drivers generated from processes, priorities, resource allocation and production–consumption patterns that result from different socio-economic development models (Oliver-Smith 1992). In essence, disaster risk drivers emanate from the ways the basic goals and parameters for growth and societal definitions of development are established and implemented (Oliver- Smith et al. 2016). But how do ongoing fundamental social processes, stemming from root causes, lead to particular ‘risk drivers’ that exacerbate existing or create new forms of risk at all levels? This is one of the main issues to clarify.

**FIGURE 3:**
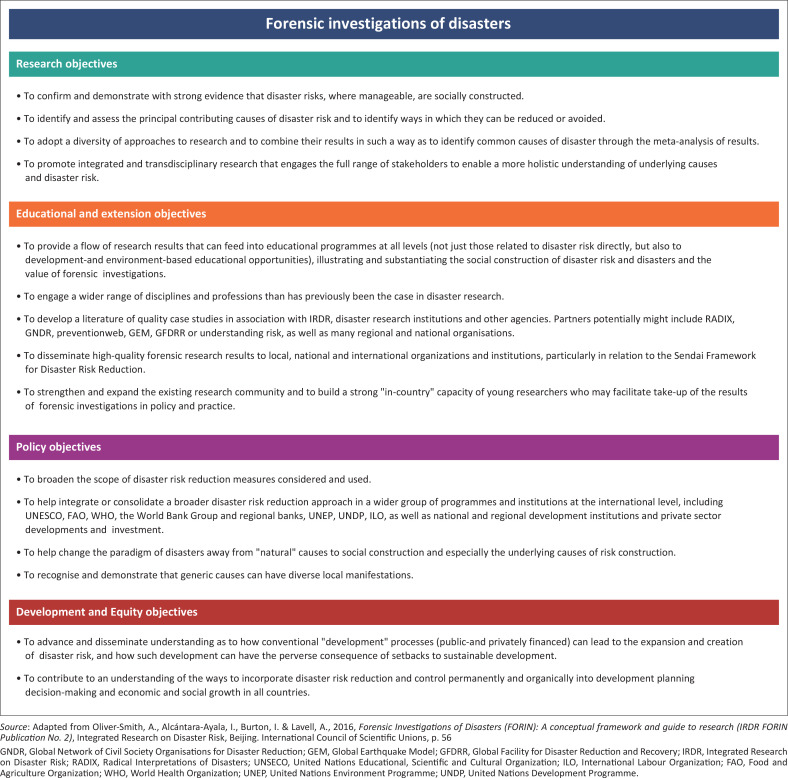
Specific objectives of Forensic Investigations of Disasters investigations.

The nature of the overarching goal of forensic investigations of disasters and risk is to identify root causes in order to assess and address them. Therefore, FORIN’s specific objectives for research, education and extension, for policy and development and for equity were set out as guidelines (Figure 3).

Four research approaches were suggested: (1) Retrospective longitudinal analysis (RLA), (2) FORIN disaster scenario building (FDSB), (3) comparative case analysis and (4) meta-analysis (Oliver-Smith et al. 2016). All four privilege a longitudinal approach. These perspectives assume a causal chain established between the patterns of loss and damage and social forces that drive the construction of risk. Forensic Investigations of Disasters (FORIN) also examines root causes, exposure and vulnerability. One way of framing research is to search for both strong and weak drivers. Furthermore, it should be clear that some root causes are more subject to management or control than others, and one purpose of forensic research is to identify causes and open pathways to their reduction or eradication through policy formulation and practice (Oliver-Smith et al. 2016).

**FIGURE 4:**
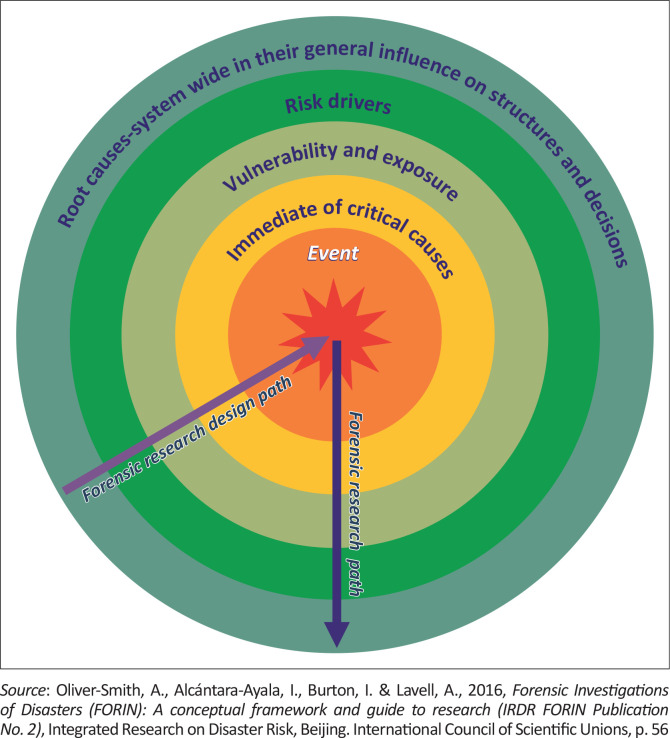
The design path of forensic disaster research and the actual path through which it proceeds.

The design path of forensic disaster research starts with root causes and their general influence on structures and decisions moving through risk drivers, vulnerability and exposure factors towards immediate or critical causes in explaining the disaster and its associated impacts. The actual path taken by much forensic research starts with the disaster event and moves outward through immediate causes to vulnerability, exposure and risk drivers, towards root causes (Oliver-Smith et al. 2016) (Figure 4). Nonetheless, there needs to be a shift such that the actual path of forensic research could also start with root causes.

Two other approaches, both drawing on the PAR model and FORIN to varying degrees, were also developed: the German Committee for Disaster Reduction (ed. DKKV 2012) scheme and the Preparing for Extreme and Rare Events in Coastal Regions (PEARL) project (Fraser 2016; Fraser, Patterson & Pelling 2016) (see Fraser 2023, this issue). Both seek to understand and correct drivers of risk and root causes where possible. Despite differences in scope and level of analysis, both the German Committee for Disaster Reduction (DKKV) and PEARL are intended to support agencies and stakeholders in disaster risk management to identify interventions that address root causes and drivers of disaster risk (Oliver-Smith 2022).

The DKKV approach generally limits analysis to the institutional level and critiques FORIN research for addressing more political macroeconomic root causes and underestimating local and context-specific drivers. By contrast, PEARL’s Risk and Root Cause Assessment (RRCA) analyses the production of risk and vulnerability in coastal regions to develop tools for forecasting, prediction, early warning systems and adaptive management strategies (Oliver-Smith 2022). Risk and root cause assessment analysis extends FORIN by refining analysis of disaster causation, including during the response and reconstruction periods. However, from a broad perspective, unlike FORIN, neither the DKKV nor the PEARL analyses of root causes advance a direct critique of the fundamental structure, organisation and priorities of the state and the global political economic system that constructs risk (Oliver-Smith 2022). Yet, the resulting analysis was nevertheless critical of land use and planning regimes in different localities, as evidenced by Fraser et al. (2020).

Forensic Investigations of Disasters represents strong elements of continuity with previous disaster risk research (Blaikie et al. 1996; Lavell & Maskrey 2014; O’Keefe, Westgate & Wisner 1976; Oliver-Smith 1998; Wijkman & Timberlake 1984). It integrates the strengths of earlier frameworks (Blaikie et al. 1996; ed. DKKV 2012; Fraser 2016; Fraser et al. 2016; Turner et al. 2003) and builds on lessons learned in practice and on the demonstrated added value of research to support disaster risk management (DRM) policy formulation. This speaks to the need to link structural analyses to policy and practice. The gap between structural analysis and practical application is wide because one approach addresses what are essentially global systems and processes and the other deals with national and local concerns (Lavell 2003). Nevertheless, the search for root causes remains a central component of what disaster risk management should be involved with; often not only as an initial recognition of the processes involved in disaster risk creation and construction (DRCC) but also to understand the interrelationships and interdependencies among disaster risk drivers, their systemic nature and challenging complexity.

## Research methods and design

### The first decade: An assessment of Forensic Investigations of Disasters 2011–2021

The word ‘forensic’ is more commonly associated with mortality, post-mortems or criminal detective work (Integrated Research on Disaster Risk 2011). The adoption of both the terms ‘forensic’ and ‘investigations’ can perhaps be considered an unfortunate choice because of the dominant association for many authorities with suggested criminality or a lack of authority. Nevertheless, the term forensic investigation has been employed in reference to systematic and analytical investigation in search of root causes and risk drivers.

The second version of the FORIN framework was formulated for maintaining the original idea adopted by IRDR to organise a series of comparative ‘in-depth’ disaster investigations, using a common methodology, to search for the root causes of disaster risk and disasters through meta-analysis. The value of such an approach was to explore the hypotheses that disasters are not simply single ‘one-off’ and ‘place-based’ events but are interconnected in at least two ways. First of all, disasters have cascading consequences. The collateral effects of a disaster can be felt in many faraway places and sometimes throughout the world. This notion is now widely referred to as the systemic nature of risk as evidenced by several recent crises: the global disaster triggered by the financial crisis of 2008–2009 and later the nuclear meltdown at Fukushima (MLIT 2011; Takeuchi & Chavoshian 2011), flooding events in Thailand and their impact on supply chains (Chongvilaivan 2012) and particularly the COVID-19 pandemic (Alcántara-Ayala et al. 2021).

The second interconnection is that very different disasters may share common underlying causes. The corollary of this fact is that the focus of national and international activities should not only be on disaster risk reduction (DRR) but also on DRCC. This has been increasingly recognised over the last 20 years particularly in Latin America (see Lavell et al. 2004; Lewis & Kelman 2012). For a long time now, disaster scholars have made the case that there is no such thing as a ‘natural’ hazard (O’Keefe et al. 1976), and more recently a number of articles and editorials (Alcántara-Ayala et al. 2021) have drawn attention to DRC and argued that disasters are 100% the result of human choices and decisions, whether made consciously or not.

### Ethical considerations

This article followed all ethical standards for research without direct contact with human or animal subjects.

## Results

Unfortunately, the anticipated meta-analysis has not been completed. The studies that have used or referred to the forensic (FORIN) approach have not used sufficiently common or similar methodologies to facilitate (or permit) a meta-analysis. The reasons for this are not completely clear. What appears to be the case, however, is that there was neither leadership nor a mechanism to provide a suitable arrangement for the selection of FORIN studies or for their oversight. In addition, no financial support was foreseen within IRDR to cover the cost of comparative ‘in-depth’ disaster studies. The FORIN guideline documents are perhaps not specific enough to generate a methodology. They do provide a general ‘guide to research’, but not a well laid out, precise and detailed case study design and method.

Here it is important to underscore the challenges of developing specific methodologies as opposed to large-scale conceptual guidelines. The variables and factors at play are so numerous and dynamic in so many different contexts and when faced with so many different hazards that developing common methods is very difficult. Some efforts at funding to cover the costs of an international set of comparative forensic studies were unsuccessfully made. The lack of success may perhaps in part be explained by concerns that the studies would be too much focussed on the identification and attribution of blame and responsibility. As mentioned earlier the word ‘forensic’ most commonly brings to mind a legalistic context and the attribution of blame. By misunderstanding the research grant applications in this way, funders likely shied away from controversial attribution of blame. This is despite the fact that the main intent of the forensic approach has been to search for general, root and systemic causes and not the attribution of blame.

A rapid survey conducted in 2020, updated in 2022, produced a bibliography of 61 publications related to FORIN. The articles can be broadly classified into nominal categories (see Table 1). Beyond the articles written by the IRDR scientific committee (e.g. Burton 2010; IRDR 2011; Oliver-Smith et al. 2016), many articles call for the continued support, development and need for FORIN (Cutter 2018; Marchezini 2020; Mejri et al. 2017). In examining hydrogeological risk perception in Southern Italy, Antronico et al. (2019) hoped that FORIN would ‘help change the mind-set of public actors, the private sector and governments, and create a more determined movement towards risk reduction and control’. In fact, they argued, ‘disaster risk reduction, control and prevention must be permanently and organically integrated into decision-making processes’.

**TABLE 1:** Classification of Forensic Investigations of Disasters related publications.

Classifier (Node)	References
Pre-FORIN papers (Developmental) before 2010	Stonich (1992); Gibbs (1996); Mitchell (ed. 1999)
FORIN Documents (Foundational)	Burton (2010, 2015); Integrated Research on Disaster Risk (2011); Oliver-Smith et al. (2016).
FORIN Discussion papers	McBean (2012); Rovins (2013); Alcántara-Ayala and Oliver-Smith (2014); Masys (ed. 2016); Prizzia (2016); Oliver-Smith et al. (2017); Alcántara-Ayala (2019); Alcántara-Ayala and Oliver-Smith (2019); Oliver-Smith (2022)
FORIN Review papers	Fraser et al. (2016, 2020)
Papers calling for a forensic methodology and FORIN	Mejri et al. (2017); Cutter (2018); Antronico et al. (2019); Marchezini (2020)
Critique of FORIN & Post disaster and forensic analysis	Vuorio et al. (2021); ÀÌ¿µ¿õ (2021)
Paper methodology based on FORIN	Dolan et al. (2016); Fra. Paleo (2019); Puente-Sotomayor, Egas and Teller (2021); Wesely (2021)
Application FORIN + additional methods or frameworks applied	Girard et al. (2014); Keating et al. (2016); Levy (2016a,b); Dominguez et al. (2021); Giuliani, De Falco and Cutini (2022)
FORIN case studies	Oliver-Smith (2010); Sagara (2011); Huang et al. (2013); Castillo (2013); Faustino-Eslava (2013); Naruchaikusol, Beckman & Mochizuki (2013); Gotangco et al. (2014); Hsin-Chi et al. (2014); Yang et al. (2014); Beckman, Mochizuki and Naruchaikusol (2015); Brenes (2016); Nakasu, Ono and Pothisiri (2017); Yuan and Liu (2018); Lavell and Brenes (2018, 2019); Wantim et al. (2018); Mendoza and Schwarze (2019); French et al. (2020); Caplan (2021); Payo Garcia et al. (2022)
Workshops and/or event papers	Alcántara-Ayala et al. (2014); Rovins, Doyle and Huggins (2014)
Planning for the future with FORIN	Galasso et al. (2021)

Note: Articles may fall within multiple classifiers. The primary node was selected for placement within the categorical analysis structure. Articles were not double coded into multiple nodes. Please see reference list of article Alcántara-Ayala, I., Burton, I., Lavell, A., Oliver-Smith, A., Brenes, A. & Dickinson, T., 2023, ‘Forensic investigations of disasters: Past achievements and new directions’, *Jàmbá: Journal of Disaster Risk Studies* 15(1), a1490. https://doi.org/10.4102/jamba.v15i1.1490 for full reference details.

FORIN, forensic investigations of disasters.

The survey upholds the ad hoc nature of FORIN literature and applications that potentially undermine the ability to provide a set of comparable studies as a basis for meta-analysis. Variations by region, disaster type and methodology can be noticed. Case studies were located in geographically dispersed places, from Hawaii (Levy 2016a) to Tuscany (Guiliani et al. 2022). In addition, the studies covered distinct disaster types from landsides (Puente-Sotomayor et al. 2021), flooding (Mendoza 2019) and typhoons (Mejri et al. 2017), along with analyses of early warning systems (Marchenzini 2020). The methodologies were also inconsistent: French and colleagues (2020) studied the root causes of El Niño-related disasters in Peru using historical analysis and stakeholder interviews. Beckman et al. (2015) conducted semi-structured interviews of government administrators and community members in Northern Thailand to ‘investigate the interrelationships between land-use changes and climate risk’. A systemic approach was used by Wantim et al. (2018) to examine the 1999 Mount Cameroon eruption. And Caplan (2021) applied the framework in a ‘postcolonial retrospective longitudinal analysis of Haiti’s 2010 earthquake’.

Meta-analysis could be carried out using studies in different regions and for different disaster types but not without a common methodology. And the methodology needs to be quite standardised and specific and detailed – and this has not been provided for FORIN so far.

Forensic Investigations of Disasters was also commonly used alongside other forensic disaster analysis frameworks. Some authors documented that this complementary use of FORIN was to strengthen the analysis. Examples include the studies using the CEDIM Forensic Disaster Approach (CEDIM FDA) (Girard et al. 2014), the Post Event Review Capability (PERC) methodology (Keating et al. 2016) and the PAR model (Giulanai et al. 2022). Another perspective argued that the use of PEARL was responding to ‘limits identified in the established FORIN approach’ (Fraser et al. 2016).

The cause of this variation of approaches was speculated upon in a Korean article examining the lack of root cause discussion (이영웅 2021). The authors observed that root cause analysis had yet to become its own discipline and inferred this was because of the multidisciplinary and ‘profound procedures’ that were ‘acting as barriers to entry for researchers’. Vuorio and colleagues (2021), FORIN instruction had only been provided at a ‘general level’, and that more guidance was needed in order to allow a ‘modern international safety investigation procedure’.

A positive way of viewing the ad hoc application of FORIN suggests that moulding its use to local situations has allowed in-depth research that has been critical post-disaster assessment. Several countries in Latin America, including Ecuador, Uruguay, Peru, Honduras, Costa Rica and Guatemala have seen FORIN used in this way in recent years. For example, three case studies were developed to promote the use of the method in the context of a United Nations Educational, Scientific and Cultural Organization (UNESCO) project. In Peru, the case study was focused on Piura and the 2015 flooding, triggered by El Niño Costero, using retrospective longitudinal analysis. In Ecuador, the case study was developed in the Imbabura province, using a comparative analysis between two landslide episodes, affecting local commerce. In Uruguay, the FORIN was applied in the cities of Bella Union and Artigas, using a combination of longitudinal retrospective and comparative analysis of the way the two cities coped with flooding episodes in 2015 (Brenes 2016).

Another study elaborates on the risk drivers behind the construction of disaster risk in Choloma, Honduras during the period following the impact of hurricane Fifi (1974). Based on the analysis of damages and losses and the risk context during the 1970s, and the social and economic processes leading to the existence of a city of over 300 000 people today (as compared with less than 10 000 in 1974). The study tries to project impact scenarios if a similar event affected Choloma under the new vulnerability scenario existing today. A similar analysis was carried out in the case of Turrialba, in Costa Rica, a secondary city located between two rivers and near an active volcano. In both cases, the hypothesis and scenarios that were postulated were verified by the impact of hurricane Eta (2020) affecting Choloma and extreme rainfall in the Costa Rica Caribbean in 2021 that triggered a flooding episode in Turrialba (Lavell & Brenes 2018). The eruption of Fuego volcano and the human impact was also analysed using retrospective longitudinal analysis. The case study focused on vulnerability construction from the 1970s to 2019 and generated arguments that help to explain the social dynamics and response during the volcano eruption and the barriers that national authorities faced during the relocation phase in the aftermath of the eruption (Lavell & Brenes 2019).

**FIGURE 5:**
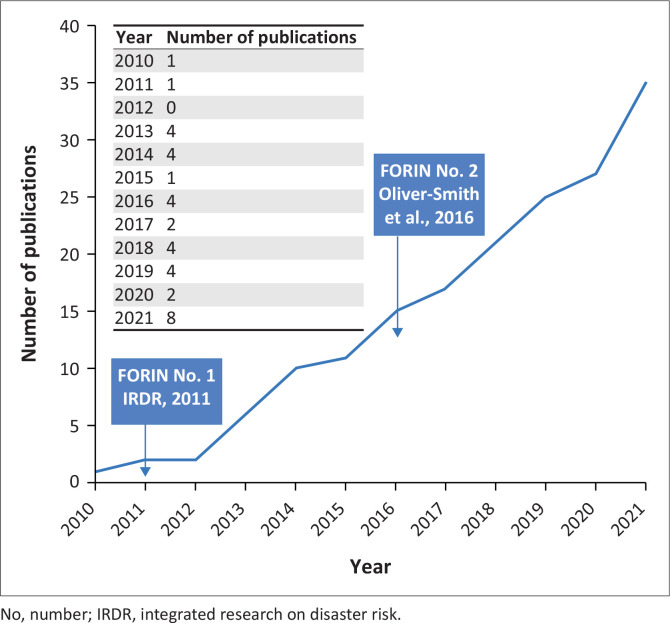
Number of articles applying Forensic Investigations of Disasters methodology (direct or indirect application) (cumulative).

The use of FORIN over time has been increasing. Figure 5 shows the cumulative growth of case studies and applications of FORIN in forensic disaster investigations over time. In 2021, there were at least eight applications of FORIN. Additionally, FORIN has been included in the United Kingdom’s Tomorrow’s Cities hub, where the decision support environment will ‘deploy state of the art … participatory methodologies and historical forensic investigations of disasters (FORIN analysis)’ (Galasso et al. 2021) (also see McDermott et al. 2022).

For instance, in Latin America and the Caribbean (LAC), both the PAR model and the FORIN approach have had a significant impact, at least from a conceptual perspective to such an extent that various governments have enthusiastically taken up FORIN since its translation into Spanish, as have international agencies and non-governmental organisations (NGO). This is the case of Uruguay, Peru, Colombia, Costa Rica (Lavell & Brenes 2018), Guatemala (Lavell & Brenes 2019) and probably in some instances Mexico, as far as governments or parts of government go (those dedicated to analysis and research and evaluation), the World Bank in Central America (Lavell & Brenes 2018), the Inter-American Development Bank and UNESCO, among others at international agency levels.

The adoption and impulse given to different methods and approaches are influenced by two issues, and in turn, these differ regionally and nationally. Firstly, expanding methods and approaches is very much related to the relations between researchers and implementation actors. In LAC, it is interesting and significant that several very top management roles in many countries are now filled by LA RED members or adepts of social construction ideas on risk and disaster (Alcántara-Ayala 2019; Lavell, Brenes & Girot 2013). Secondly, however even where it is clear that the whole issue of responsibility and the causal process is well accepted in the discourse and in research results, there is no guarantee that underlying root causes are actually addressed given the location of decisions in the hands of dominant economic and social actors who many times may even derive benefit from DRCC. Nevertheless, within such limits, it could be argued that recognition of root causes and their connectivity to risk drivers can lead relevant DRR stakeholders to explore alternative positions in addressing the complex interactions that shape the social construction of risk within the mosaic of skewed, class-biased development practice that continues to affect all societies.

That being the case, it is clearly essential that unravelling the processes of social construction of risk and disasters (Burton 2010, 2015), and particularly recognising root causes (Oliver-Smith et al. 2016, 2017a, 2017b) remains a challenge. Even after some years of its inception and partial acceptance, especially in LAC, FORIN remains poorly visible in some policymaking arenas. One indicator of the continued peripheral status of forensic investigations of disasters is the lack of presence in research agendas and policy-formulation beyond LAC. This may be explained by the generation of many more innovative ideas on risk and disasters in LAC than in many other more ‘conservative’ regions, so concern about FORINs’ lack of visibility and application in the wider world is justified.

The authors strongly believe that in the face of the great problems posed by disasters and the DRCC, such as the global disaster triggered by the COVID-19 pandemic, FORIN should be used routinely to underpin policy-informed development and planning. Legacy aspects of research on disaster risk construction and policy issues, as well as efforts of diverse stakeholders should be directed towards strengthening human and institutional capacities to manage disaster risk from an integrated approach. An update of the approach of FORIN and the design of a new stage on disaster risk need to be sought. In such an effort, a new ‘framework’ should be established that involves a more radical rethinking of what is needed in the new era of climate change and pandemics and the need for far-reaching transformation. The central problem is not the research *per se*. The biggest problem is how to transfer knowledge into the political decision-making sphere and how to bring a global rethink politically, and that is the greatest challenge FORIN faces.

## Discussion

### Questions and ideas for future Forensic Investigations of Disasters research

#### Harvesting the Forensic Investigations of Disasters results

As previously noticed, the application of a forensic approach (FORIN) in disaster risk research has fallen short of early hopes and expectations. There is much more to be performed. This should not deter efforts to harvest and make use of the results that have been achieved so far. One idea that is being explored is the preparation of two books or reports. The first is the publication of a collection of completed FORIN studies. There are at least 20 of these studies, as listed in Table 1. A small group of editors is being formed to put the studies together in one volume. For this purpose, some may need to be reduced in length and detail. Others may be short and concise enough to be included completely. Where a shorter version is needed the author, or authors, of the case study will be asked to prepare it. Tasks for the editors of the book of case studies will include the preparation of an introduction and conclusion and explanation of its purpose and also the grouping or ordering of the articles by types of hazard event, regional location and the manner and extent of drawing upon the FORIN framework.

Initially it was hoped and expected to make a meta-analysis of the case studies in order to extract a set of common conclusions and results. This is not possible at a level rigorous enough to qualify as a valid meta-analysis because the case study researchers have not had the benefit of a sufficiently detailed and common methodology. Nevertheless, it should be possible to carry out a loose and qualitative collective analysis of the articles. This would be the content of a second book or report, or perhaps a concluding chapter of the first book – the collection of articles. This collection and analysis and harvesting of the case studies will provide a step towards the next phase in the development of FORIN and the further and improved use of a forensic approach.

#### The next phase of forensic investigation

One possible and potentially important feature of the next phase of FORIN or the forensic approach is developing a more precise and specific set of guidelines that could be followed closely and rigorously enough to permit a cross-cutting analysis that would qualify as a meta-analysis. This should not be limited to a simple set of case studies in the way that has been carried out so far. Any empirical studies should be set in the context of a wider range of risks beyond those used previously. This is because it is now understood that investigations into the root and common causes of disasters can and should include, if not a full range of all kinds of disasters, then at least a much wider range than previously attempted. This can include the effects of climate change, the COVID-19 pandemic and any future pandemics, the rise in international conflicts and tensions, and other possible crises both identified and not yet known about.

Proposals are now being developed, meetings are being planned whereby experts, research agencies and financial supporters can be brought together with policy analysts to formulate the design of the next phase of FORIN (forensic) studies. At first, these are being developed by independent researchers, but an expanded role for intergovernmental agencies, such as the United Nations Office for Disaster Risk Reduction (UNDRR) and its regional components, is possible and needed. Such work is required urgently in order to feed in a timely fashion into negotiations on the follow-up to the Sendai Agreement, which expires in 2030. Just as the Intergovernmental Panel on Climate Change (IPCC) preceded the drafting and agreement of the United Nations Framework Convention of Climate Change (UNFCCC) so too must the next phase of forensic investigations be linked into an Intergovernmental Panel on Disaster Risk (IPDR). Consultations and initiatives must begin as quickly as possible with inputs from FORIN.

The changing nature of the disaster crisis must not be overlooked. It is more than the need to consider a wider and if possible, complete range of disaster risks. It must be recognised that this is now an ongoing process of what is being called a poly-crisis. Not only are there more risks and disasters, but there is an emerging pandemic of crises. The formulation of the IPDR and the follow-up to the Sendai Agreement have to recognise these new circumstances and take them into account.

## Conclusion

Moving the FORIN framework forward requires renewing interest in the institutional process, and thus governance, and in the way DRM is considered in national and international planning. This implies necessary insistence on the fact that disaster risk and disasters are not just themes in themselves but rather are acute reflections of the breakdown in many basic principles of a secure life on this planet. Our concern also points to the question, what would become of a society in which disaster risk does not have its rightful place? Contributions are needed at the global level on how and why risks are created and constructed (Maskrey et al. 2021), as well as how and why disasters materialise in society (Alcántara-Ayala et al. 2021). There is also a responsibility to make sure disasters and the risk that precedes them are not seen as silos but as critical elements to be avoided in achieving increased sustainability, human rights and accountability in the frame of development and human security. The call for systemic thought, integrated, holistic approaches (Cardona 2004), getting disaster risk reduction (DRR) into the deoxyribonucleic acid (DNA) of organisations is imperative but far from being a reality as mainstreaming arguments and approaches persist, and DRM is still seen to be a sector among others (Lavell & Maskrey 2014).

In reaction to skewed development and malgovernance (Lavell & Maskrey 2014), the world is beginning to experience a transition to a new paradigm of human thriving and earth care. Accordingly the whole idea of governance for DRM or for risk should be reconsidered and emphasis be placed on governance for sustainability, where this is informed not only by disaster risk but also in an integrated manner by many other so-called mainstreamed or cross-cutting thematic concerns. For example, the essential elements of social democracies not only nationally but also internationally and globally: primacy of social justice for all, equality and access to healthcare, education and security.

The authors continue to believe that such developments can be facilitated by creating an IPDR. Such a body might be modelled upon the IPCC and its role in support of the UNFCCC. It would consist of government-nominated experts who would make periodic assessments of the science of disaster risk (Cutter et al. 2015) (DRR and DRCC), and it would have a similar mandate of being policy-relevant and not policy prescriptive. Given recent global developments with the COVID-19 pandemic, climate change and increasing frequency of disasters, the time seems urgent for a change. Indeed, with the various international agendas at play and their interconnection and interdependencies, the root causes approach seems well-placed to promote that.

Along this vein, it is worth observing that the IPCC was established in 1989 – 3 years before the first agreement on the Framework Convention on Climate Change (UNFCCC 1992). And that the first report of the IPCC included proposals and ideas for the UNFCCC, which were used and taken up in Rio in 1992. So, the new IPDR or equivalent should be created and move quickly to put ideas on the table well prior to the renewal or replacement of the Sendai Agreement in 2030 (only 7 years away). Putting transdisciplinary alternative ideas on the table, we should have the reassurance that would not violate the fundamental requirement of the IPCC or the IPDR to be ‘policy-relevant and not policy prescriptive’.

Since the conception of FORIN (2010), the world context has changed dramatically. The impacts of climate change have become evident, immediate and more threatening. The global COVID-19 pandemic has resulted in more than 6.8 million deaths (10 March 2023) that reveal underlying inequalities. Geopolitical tensions have increased because of Russia’s invasion of Ukraine as well as the challenge of energy conversion from fossil fuels to renewables. There are increasing doubts about long-term food and water security.

The authors recognise that there is certainly a requirement (and requirements) for new approaches and understanding of disaster risk and disaster risk research considering: (1) the incomplete ambitions and failures of FORIN and the forensic approach; (2) the ongoing transformational changes exemplified by climate change, the COVID-19 pandemic, the threats and manifestations of global international confrontations and conflicts and (3) the fact that nation-states (both social democracies and more authoritarian regimes) are increasingly adopting short-term self-interested policies and actions (in some places this is referred to as ‘populism’), at the very time when the ongoing risks and crises and associated disasters are global and in need of international and worldwide collective response and management.

Treatment of disasters as phenomena separate from these other issues no longer makes sense. The changes in the global context require a more holistic approach to disasters. It is no longer appropriate to refer to ‘disasters’ or ‘disaster risk’ in isolation, but, rather, we need to think in aggregate terms of the problems and the transitions that the world situation now requires. In this context, it is not sufficient only to plan for the continuation and revision of future FORIN research and the pursuit of forensic investigations. What is needed to help guide and shape such research and investigations is creative imagination and leadership. The worldwide academic, research and intellectual communities need to imagine and create ideas of how to proceed in these times. The authors expect in a subsequent article to help stimulate such efforts and make some initial contributions to research planning and creative thinking, considering the interactions and interdependencies among international agendas from a transdisciplinary approach to properly address the developmental nature of disaster risk drivers.

To put it succinctly, what is needed above all is: (1) to stop treating disasters associated with extreme events as only separate and isolated, and move to an ‘all risks’ or ‘all disasters’ perspective and framework and/or paradigm; (2) to begin to envision global transformation(s); and the creation of a new and better world order, to avoid moving further into a global existential crisis; and (3) to propose and develop an (IPDR modelled on the IPCC). This is not a task for a few individuals. It is a challenge for us all.

## Data Availability

The authors confirm that the data supporting the findings of this study are available within the article.
